# The Spectrum of Magnetic Resonance Enterography Findings and the Role of Diffusion-Weighted Imaging in Patients with Active Crohn’s Disease

**DOI:** 10.34172/mejdd.2024.364

**Published:** 2024-01-31

**Authors:** Arvin Arian, Ghazal Roostaei, Seyede Sahel Rasoulighasemlouei, Foroogh Alborzi Avanaki, Nasser Ebrahimi Daryani

**Affiliations:** ^1^Department of Internal Medicine, Imam Khomeini Hospital Complex, Tehran University of Medical Sciences, Tehran, Iran

**Keywords:** Magnetic resonance enterography, Active Crohn disease

## Abstract

**Background:**

The goal of this study was to evaluate magnetic resonance enterography (MRE) findings and assess the role of diffusion-weighted imaging (DWI) in patients suffering from active Crohn’s disease.

**Methods:**

This retrospective study included a total number of 76 patients diagnosed with active Crohn’s disease, as established by the Crohn’s Disease Activity Index (CDAI). The study consisted of 30 women and 46 men, ranging in age from 13 to 72 years. All participants underwent MRE with DWI sequences. The study was conducted at Imam Khomeini hospital in Tehran between 2013 and 2018. The imaging modality utilized for the study included a 3-T SIGNA Excite MRE machine and a Siemens Magnetom 3-T magnetic resonance imaging (MRI) machine.

**Results:**

Bowel wall restriction was observed in less than half of the patients, and no significant correlation was found with extramural findings such as mesenteric edema. The study did not reveal any meaningful association between diffusion restriction and specific mural enhancement patterns, mesenteric lymphadenopathy with or without enhancement, or the length of the affected segments (*P*>0.05). The most common findings observed in MRI were ileum thickness in 72.4% of patients, mesenteric lymphadenopathy without enhancement in 46.1%, ileocecal thickness in 42.1%, DWI findings in 42.1%, the presence of a comb sign in 36.8%, and jejunum thickness in 30.3% of patients.

**Conclusion:**

MRE findings are useful in the evaluation of Crohn’s disease activity.

## Introduction

 Crohn’s disease is a chronic inflammatory bowel disease that can occur throughout the gastrointestinal tract, often accompanied by various complications. Perianal complications, such as perianal fistulas, fissures, and abscesses, are observed in approximately 26% of patients.^1^ Consequently, patients with Crohn’s disease undergo multiple imaging and diagnostic studies throughout the disease course, including upper endoscopy, colonoscopy, capsule endoscopy, barium studies, computed tomography (CT) scans, and magnetic resonance imaging (MRI).

 Ileo-colonoscopy is commonly performed for tissue sampling and disease confirmation in conjunction with the assessment of symptoms and laboratory tests. However, its access to the small bowel is limited. Video capsule endoscopy was introduced to visualize the small bowel directly; however, it is contraindicated in patients with strictures and obstructions. Furthermore, endoscopic studies have limitations in evaluating submucosal and serosal abnormalities, as well as extramural manifestations, despite their efficacy in assessing ulcers and mucosal lesions. Barium studies are less frequently used due to their poor sensitivity and specificity.^2^

 CT scan is commonly performed for evaluating Crohn’s disease, but the cumulative radiation exposure associated with multiple exams raises concerns.^3^ MRI remains a radiation-free alternative to CT scans, offering comparable sensitivity and specificity in diagnosing small bowel involvement.^4^ Initially, MRI with enteroclysis was performed, but subsequent studies demonstrated that magnetic resonance enterography (MRE) has similar efficacy, except for the assessment of superficial ulcers, which are better evaluated using MR enteroclysis. MRE is preferred over enteroclysis due to its less invasive nature.^5,6^

 Multiple imaging studies are conducted throughout the disease course to evaluate the disease extent and complications, which are crucial for patient management and treatment planning. In this study, our aim was to assess the findings of MRE in patients with pathologically confirmed Crohn’s disease who were clinically experiencing disease flare-ups.

## Materials and Methods

###  Subjects and the Study Design

 This observational case series study received ethical approval from the Ethics Committee of Tehran University of Medical Sciences, with approval ID: IR.TUMS.IKHC.REC.1397.374. Written consent forms were obtained from all patients included in the study. The decision to perform MRE was made by the referring clinicians for the clinical management of the patients, and no additional interventions were conducted by the Radiology Department specifically for research purposes.

 Cases of Crohn’s disease confirmed with pathology in the flare phase based on clinical presentations, Crohn’s Disease Activity Index (CDAI), and serological markers such as CBC, CRP, and stool calprotectin, who were referred to the imaging center of our hospital between 2013 and 2018, were included in this study. Data regarding age, sex, various imaging features of the bowel loops, extramural findings, and any potential complications were recorded.

###  Patient Preparation and MRE Technique

 A low residue diet for 2 days and fasting 6 hours before the imaging was recommended to reduce fecal matter in the colon and improve oral contrast tolerance, respectively.^2^

 For the study, 1500 cc of the polyethylene glycol (PEG) solution with a concentration of 58.96 g/L was administered orally to each participant. The initial dose of 1500 cc was given 45 minutes before the study, followed by an additional 500 cc every 15 minutes. The PEG solution served as a biphasic contrast agent, characterized by low signal intensity in T1-weighted images and high signal intensity in T2-weighted images. This allowed for a better evaluation of mural enhancement after the administration of intravenous contrast.

 The use of a high volume of oral contrast aimed to achieve optimal distension of the small bowel. To confirm the adequacy of small bowel distension, a coronal T2-HASTE image was obtained. If the distension was deemed insufficient, an additional 500 cc of oral contrast was administered. However, if the desired distension was still not achieved, the patient was subsequently excluded from the study.

 As an intravenous (IV) contrast agent, 10 cc of 0.5 mmol/mL solution of gadopentetate dimeglumine was used.

 All imaging was performed using the 18-channel body coil on a Siemens Magnetom 3-T MRI scanner. The MRE protocol performed in this study is detailed in Table 1. Following the administration of intravenous contrast, volumetric interpolated breath-hold (VIBE) examination images were acquired immediately and then repeated at 30-, 70-, and 180-second intervals.

**Table 1:** MRE Protocol

	**Plane**	**Section width (mm)**	**Field of view (mm)**	**TR/TE (ms)**	**Matrix**	**Flip angle**
HASTE	Coronal	5	400*450	1500/92	227*384	
HASTE	Axial	5	400*450	1500/92	227*384	
HASTE	Sagittal	5	400*450	1500/92	227*384	
True-FISP	Coronal	5	350*400	3.9/2	230*256	60
HASTRIM	Coronal	5	400*450	1350*82	248*384	150
VIBE	Coronal	3	400*480	4.8/2.3	177*320	10

Abbreviations: HASTE, T2 weighted half-fourier single-shot turbo spin-echo; True-FISP, True fast imaging in steady-state precession; VIBE, Volumetric interpolated breath-hold examination.

###  Radiological Analysis

 The analysis of the images was conducted by an expert gastrointestinal radiologist with over 5 years of experience in interpreting MREs. The radiologist examined various imaging features of the bowel loops, including bowel wall thickness, patterns of mural enhancement, and diffusion restriction. Additionally, extramural findings such as mesenteric edema, comb sign, lymphadenopathy, peritoneal thickening, ascites, as well as the presence of fibro-stenotic bowel segments and complications (e.g., abscesses and different types of fistula formations) were assessed.

 Bowel wall thickness greater than 3 mm was considered abnormal according to established criteria.^7^ In order to evaluate bowel wall enhancement and restriction, the adjacent normal bowel loop was used as a reference.^8^ Lymph nodes were categorized into enhancing and non-enhancing groups, with a 10-mm cutoff considered for the non-enhancing group.^9^ Criteria for identifying fibrostenosing segments included low T2 signal intensity, minimal enhancement, and increased bowel wall thickness.^9^

###  Statistical Analysis

 The categorical data were presented as frequency (percentage). The association between different variables was assessed using the chi-square/Fisher’s exact test, and a *P *value less than 0.05 was considered statistically significant. The data were analyzed using IBM SPSS software version 26.

## Results

 The data were collected and analyzed from 76 patients with established Crohn’s disease who were suspected to be in the active phase. Diagnosis of Crohn’s disease was established based on radiological, endoscopic, and histological findings in a patient with compatible clinical presentation. We used CDAI and serological markers like complete blood count (CBC) and stool calprotectin to describe disease activity.

 The mean age was 37.04 ± 14.71, ranging from 13 to 72 years old. Out of the 76 patients included in the study, 46 (60.5%) were men, and no significant correlation was observed between sex and age distribution. Bowel wall thickening was detected in all cases on non-contrast-enhanced images. The frequency of small bowel, ileocecal, and colonic wall thickening was 78.9% (60 out of 76), 42.1% (32 out of 76), and 20% (15 out of 76), respectively, as shown in Table 2. Rectal wall thickening was observed in one patient, as was duodenal wall thickening, and in both cases, there was involvement of other segments of the bowel. Among the 55 patients with ileal wall thickening, 18 also exhibited concurrent jejunal wall thickening. Isolated jejunal wall thickening without involvement of the ileum or ileocecal region was identified in only one patient.

**Table 2:** MRI findings (Mural findings)

**Bowel wall thickening**	**Yes**		**No**	
	**Count**	**Row valid N %**	**Count**	**Row valid N %**
Duodenal wall thickening	1	1.3%	75	98.7%
Jejunal wall thickening	23	30.3%	53	69.7%
Ileal wall thickening	55	72.4%	21	27.6%
Ileocecal wall thickening	32	42.1%	44	57.9%
Cecal wall thickening	3	3.9%	73	96.1%
Ascending colon wall thickening	3	4.0%	72	96.0%
Transverse colon wall thickening	3	3.9%	73	96.1%
Descending colon wall thickening	4	5.3%	72	94.7%
Sigmoid colon wall thickening	10	13.2%	66	86.8%
Rectal wall thickening	1	1.3%	75	98.7%

 All patients in the study showed mural enhancement after contrast administration. Among them, 56 patients displayed transmural enhancement, while the remaining patients showed a stratified pattern of enhancement.

 Diffusion restriction of the bowel wall was observed in 42% of the cases. Fibrostenosing disease was identified in 29 patients. The frequency of other extramural findings, such as fibrofatty proliferation, mesenteric lymphadenopathy, peritoneal thickening, comb sign, and ascites, can be found in Table 3.

**Table 3:** MRI Findings (extramural findings)

**MRI findings**	**Yes**		**No**	
	**Count**	**Row valid N %**	**Count**	**Row valid N %**
Fibrofatty proliferation	21	27.6%	55	72.4%
Mesenteric lymphadenopathy with enhancement	15	19.7%	61	80.3%
Mesenteric lymphadenopathy without enhancement	35	46.1%	41	53.9%
Peritoneal thickening	2	2.6%	74	97.4%
Comb sign	28	36.8%	48	63.2%
ascites	4	5.3%	72	94.7%

 No significant correlation was found between diffusion restriction and specific mural enhancement patterns, mesenteric lymphadenopathy with or without enhancement, or the length of the involved segments (*P* > 0.05).

 Among the 28 patients with a comb sign, only half of them showed diffusion restriction. Similarly, only one out of the eight patients with mesenteric edema showed diffusion restriction. These findings indicate a lack of clinically significant correlation, as determined by Fisher’s exact test.

## Discussion

 Crohn’s disease is a chronic and inflammatory condition that can affect various segments of the gastrointestinal tract, from the oral cavity to the rectum. This disease primarily affects individuals in their younger ages, necessitating long-term management. Consequently, the identification and evaluation of acute and chronic flares are necessary in formulating an effective treatment regimen for patients.^10^ Accordingly, the establishment of an appropriate diagnostic approach for assessing acute flares assumes important significance.

 According to the comprehensive study conducted by Ahmad and colleagues in 2021, it was concluded that MRE exhibited a sensitivity of 79.25% and specificity of 74.25% when assessing the activity of Crohn’s disease. These findings suggest that MRE emerges as a suitable modality for evaluating acute flares of Crohn’s disease.^11^

 MRE has emerged as a highly effective approach for evaluating the activity of Crohn’s disease, offering numerous advantages. Firstly, it eliminates the need for radiation exposure while providing excellent soft tissue contrast and the ability to visualize gastrointestinal structures comprehensively. With its multiplanar capability, it enables a thorough assessment from various angles and facilitates the detection of both mural and extramural complications. Moreover, MRE excels in defining the disease activity delivering detailed imaging results. Additionally, it can be combined with perianal imaging, further enhancing its diagnostic capabilities. The collective benefits of MRE make it a valuable tool in identifying the activity of Crohn’s disease.^12^

 Furthermore, previous studies have indicated that active inflammation of the intestinal wall can result in diffusion limitation observed in MRE with diffusion-weighted imaging (DWI) sequences. A study conducted in 2019 involving 54 patients diagnosed with Crohn’s disease supported this observation, revealing a significant decrease in the apparent diffusion coefficient (ADC) values within the affected segments of the intestine compared with the unaffected segments.

 Mean ADC values [1.5 ± 0.4 (0.9–2.5) vs. 1.2 ± 0.3 (0.6–1.8)].^13^

 In a study conducted by Aryan and colleagues involving 30 patients experiencing a flare-up of Crohn’s disease, MRE was a suitable diagnostic tool following colonoscopy. The study concluded that MRE exhibited an average sensitivity and high specificity in detecting Crohn’s disease-related lesions in the colon. Additionally, MRE demonstrated a high sensitivity and average specificity in identifying ileal lesions, making it a valuable and comparable alternative to colonoscopy.^14^

 In another study conducted by Ninivaggi et al, 60 patients with confirmed Crohn’s disease underwent MRE with DWI. The study focused on calculating the ADC values for both the involved segments (pADC) and normal segments (naADC) of the gastrointestinal tract in these patients. The results of the study revealed that the pADC values were significantly lower than the naADC values, indicating a statistically significant difference.^12^

 In a 2018 study conducted by Lee et al in South Korea, 173 patients with Crohn’s disease participated, with 61 of them classified as being in remission and 112 as having active disease. The study aimed to evaluate the role of MRE in assessing inflammation and its association with disease prognosis. The results revealed that active inflammation was observed in 93 patients (83%) with active Crohn’s disease and in 44 patients (72.1%) with remission based on MRE findings. However, no significant difference was found between these two groups. Multivariable analysis indicated that the presence of active inflammation, as determined by MRE, increased the risk of disease flare in patients. Furthermore, MRE findings of active inflammation were significantly correlated with a higher likelihood of hospitalization in patients with active Crohn’s disease. Therefore, the presence of active inflammation detected through MRE is associated with a poor prognosis in both patients in remission and those with active Crohn’s disease.^15^

 In a 2016 study by Rajabi and colleagues in Canada, MRE findings of 62 patients with Crohn’s disease were evaluated. The study identified 25 different MRE findings, with an average of 3.7 findings per MRE. The 10 most common findings included intestinal wall thickening in 54.5% of patients, post-gadolinium enhancement in 45.5% of patients, reactive lymph nodes in 31.8% of patients, luminal stricture in 27.3% of patients, increased submucosal enhancement in 27.3% of patients, increased mesenteric vascularity in 22.7% of patients, fat stranding in 18.2% of patients, creeping fat in 18.2% of patients, proximal intestinal dilation in 18.2% of patients, and stricture of bowel loops in 18.2% of patients.^16^

 In a 2016 study by Radmard et al in Iran, the MRE findings of 300 out of 594 patients with Crohn’s disease were analyzed. The study revealed that the most common phenotype observed was the inactive form, presented in 162 patients (54%). Strictures were identified in 44 patients (14.7%), while the active form was seen in 40 patients (13.3%). The number of patients diagnosed with Crohn’s disease increased from 51 in the first 6 months to 165 in the second 6-month period, indicating the promising role of MRE in identifying the predominant disease phenotype.^17^

 Based on the aforementioned data and the significance of MRE and DWI sequences in identifying the activity of Crohn’s disease, the present study was conducted on 76 participants suffering from Crohn’s disease.

 The present study shows that the most common MRE findings in patients with Crohn’s disease includes:

 Ileum thickness in 72.4%, mesenteric lymphadenopathy without enhancement in 46.1%, ileocecal thickness in 42.1%, DWI in 42.1%, Comb sign in 36.8%, jejunum thickness in 30.3%, fibrofatty proliferation in 27.6%, mesenteric lymphadenopathy in 19.7%, sigmoid colon thickness in 13.2, ascites in 5.3%, descending colon thickness in 5.3%, ascending colon thickness in 4%, cecum thickness in 3.9%, transvers colon thickness in 3.9%, peritoneum thickening in 2.6%, rectal thickness in 1.3%, and duodenal thickness in 1.3% of the patients.

 As in other studies mentioned in Table 4, bowel wall thickening and lymphadenopathy were the most common findings.

**Table 4:** Comparison of MRE findings between our and other studies

	**Our study**	**Rajabi et al** ^16^	**Onay et al** ^18^
Mesenteric lymphadenopathy	65%	31.8%	76%
Comb sign	36%	22.7	43%
Mural thickening	72%	54.5	32%
Fibrofatty proliferation	27.6	18.2	36%
Ascites	5.3	7.6	5.3%

 In active Crohn’s disease, all patients exhibited bowel wall thickening and mural enhancement. The majority of patients had involvement of the ileal and ileocecal walls. Bowel wall restriction did not correlate significantly with extramural findings such as mesenteric edema. Additionally, there was no significant correlation between diffusion restriction and specific mural enhancement patterns, mesenteric lymphadenopathy, or length of the affected segments (*P* > 0.05).

 Only half of 28 patients with comb sign and only one of the eight patients with mesenteric edema showed diffusion restriction, showing no clinically significant correlation using Fisher’s exact test.

 One of the limitations of this study was the retrospective evaluation of the files, some of which are not fully completed or do not answer the research questions. Another limitation was the number of patients. Some features cannot efficiently be studied using this method and may not appropriately represent the wider population. In addition, it was difficult to compare different cases using this method.

 For future studies, it is suggested to further research the role of DWI in determining the progression of Crohn’s disease in order to examine the treatment response and choose a better treatment plan for patients suffering from Crohn’s disease.

## Conclusion

 MRI is a valuable tool for diagnosing the active phase of Crohn’s disease, particularly by assessing bowel wall enhancement and thickening in the ileocecal area. Additionally, MRI is useful in identifying extramural findings associated with the disease.
